# Breast Self-Examination (BSE) Education: An Auxiliary Preventive Measure or a Source of Anxiety?

**DOI:** 10.7759/cureus.111064

**Published:** 2026-06-17

**Authors:** Dimitra Tsintsiou, Michael Kourakos, Maria Lavdaniti, Theodora Kafkia

**Affiliations:** 1 Nursing, International Hellenic University, Thessaloniki, GRC; 2 Nursing, University of Ioannina, Ioannina, GRC

**Keywords:** breast self-examination (bse), educational process, health anxiety, health locus of control, self-efficacy

## Abstract

Introduction

Breast self-examination (BSE) is promoted as a component of breast self-awareness and a preventive measure, yet its psychosocial impact remains debated. This study aimed to evaluate the effects of BSE education on self-efficacy, health anxiety, and health locus of control among Greek women.

Materials and methods

A prospective cohort of 298 women (mean age 48.7 ± 10.7 years) participated in a structured BSE educational intervention. Psychosocial parameters were assessed at baseline, 6, and 12 months using validated scales: general self-efficacy, health anxiety, and multidimensional health locus of control. Statistical analyses included repeated-measures ANOVA, post hoc comparisons, and regression modeling to explore temporal changes and predictors.

Results

BSE practice increased markedly from 44.3% (n = 132) at baseline to 92.2% (n = 271) at six months and 85.0% (n = 250) at 12 months (Cochran’s Q = 188.96, p < 0.001). Self-efficacy and health anxiety scores remained stable over time (p = 0.570 and p = 0.955, respectively). Health locus of control subscales revealed small but statistically significant declines in internal control (baseline: 19.2 ± 4.9; 12 months: 18.1 ± 4.6; p < 0.001) and in attribution to “powerful others” and “chance” (p = 0.040 and p = 0.039). Regression analyses indicated that prior scores were the strongest predictors of 12-month outcomes, with age associated with greater reliance on “powerful others” (β = 0.107, p = 0.040). No increase in health anxiety was observed despite the rise in BSE practice.

Conclusions

BSE education significantly enhanced preventive behavior without increasing health anxiety or altering general self-efficacy. These findings support BSE education as a complementary strategy for breast health awareness, emphasizing its role in early care-seeking rather than as a substitute for formal screening.

## Introduction

Breast cancer remains the most common malignancy among women worldwide and a leading cause of cancer-related mortality [1]. Early detection through organized screening programs, particularly mammography, has been shown to reduce mortality and improve outcomes [2,3]. However, in many settings, barriers to timely diagnosis persist, including limited access to screening, lack of awareness, and delays in seeking medical evaluation for symptoms [4].

Breast self-examination (BSE) has historically been advocated as a simple, low-cost method for breast self-awareness. It empowers women to recognize changes in their breast tissue and seek prompt evaluation. While BSE is no longer recommended as a stand-alone screening tool due to lack of evidence for mortality reduction and concerns about potential harms such as false positives and unnecessary biopsies [2,5], current guidelines emphasize the importance of breast self-awareness, encouraging women to be familiar with the normal appearance and feel of their breasts and to report any changes without delay [3].

Beyond clinical outcomes, the psychosocial impact of BSE education warrants careful consideration. Educational interventions (use of models, demonstration, and leaflets) may influence not only preventive behaviors but also psychological parameters such as self-efficacy, health anxiety, and beliefs about health control [6,7]. Self-efficacy, defined as an individual’s belief in their capacity to execute behaviors necessary to produce specific outcomes, is a recognized determinant of health behavior adoption and maintenance [8]. Health locus of control reflects the extent to which individuals attribute health outcomes to internal factors, powerful others, or chance and has been linked to engagement in preventive practices and psychological well-being [9]. Health anxiety, or the degree of worry about one’s health, may be affected by screening-related interventions, with potential implications for quality of life [10].

Despite the widespread implementation of BSE education, evidence regarding its effect on psychosocial outcomes remains limited and mixed [11,12]. Some studies suggest that education can enhance knowledge and increase BSE practice without increasing anxiety, while others highlight the risk of psychological distress associated with heightened vigilance or ambiguous findings [11,12]. Furthermore, the dynamic interplay between self-efficacy, locus of control, and health anxiety in the context of preventive interventions is not fully understood, particularly in populations with varying access to organized screening.

The present study aimed to evaluate the longitudinal effects of a structured BSE educational intervention on key psychosocial parameters, such as self-efficacy, health anxiety, and health locus of control, among Greek women. By assessing changes over a 12-month period, the study sought to clarify whether BSE education supports preventive behavior without adverse psychological consequences and to explore the evolution of health beliefs in this context.

## Materials and methods

Study design

This was a prospective cohort study designed to assess the impact of a structured BSE educational intervention on key psychosocial parameters among Greek women. Assessments were conducted at baseline, six months, and 12 months following the intervention.

Setting and participants

The study was conducted in Thessaloniki, Greece, with recruitment from community and healthcare settings. A total of 298 women participated (mean age: 48.7 ± 10.7 years). The majority resided in Thessaloniki (82.2%, n = 245), with smaller proportions from rural areas (9.4%, n = 28) and other cities (8.4%, n = 25). Educational backgrounds ranged from secondary education to doctoral degrees, and most participants were married (69.1%, n = 206). Participants were required to be adults able to provide informed consent, read and write Greek, and without a prior diagnosis of breast cancer. The exclusion criteria were cognitive impairment or inability to complete questionnaires, history of breast cancer or ongoing treatment for malignancy, and incomplete baseline data.

Intervention

All participants received a standardized educational session on BSE, delivered by a trained health professional (nurse/midwife). The 30-minute session included information on breast cancer risk, the importance of early detection, a demonstration of the BSE technique, and guidance on recognizing abnormal findings. Educational material (leaflets with the correct procedure in Greek) was provided for home reference. The intervention aimed to enhance breast self-awareness and empower women to perform BSE regularly.

Data collection

Data were collected at three time points: baseline (prior to intervention), six months, and 12 months post-intervention. At each assessment, participants completed a battery of validated self-report questionnaires:

The General Self-Efficacy Scale assesses perceived ability to cope with a variety of difficult demands (10 items; 4-point Likert scale; total score range: 10-40). The Health Anxiety Scale measures worry and concern about personal health (5 items; 5-point Likert scale; total score range: 5-25). The Multidimensional Health Locus of Control (MHLC) Scale assesses beliefs about control over health outcomes across three dimensions: internal, powerful others, and chance (18 items; 6-point Likert scale; subscale scores range from 6 to 36).

Additional data on sociodemographic characteristics, medical history, and preventive behaviors (including BSE practice, frequency, and imaging modality use) were collected through structured questionnaires.

Statistical analysis

Descriptive statistics were used to summarize participant characteristics and baseline measures. Internal consistency of the scales was evaluated using Cronbach’s alpha. Temporal changes in psychosocial parameters were analyzed using repeated-measures ANOVA with a Greenhouse-Geisser correction when sphericity was violated. Post hoc pairwise comparisons were performed with a Bonferroni adjustment. Categorical variables (e.g., BSE practice rates) were compared across time points using Cochran’s Q test and McNemar’s test for paired proportions. Ordinal data (e.g., BSE frequency) were analyzed with Friedman’s test and post hoc Wilcoxon signed-rank tests. Pearson’s correlation coefficients were calculated to explore associations between psychosocial variables. Multiple linear regression models were constructed to identify predictors of 12-month outcomes, including prior scores and demographic factors. Statistical significance was set at p < 0.05. Analyses were performed using SPSS Statistics version 27.0 (IBM Corp. Released 2020. IBM SPSS Statistics for Windows, Version 27.0. Armonk, NY: IBM Corp.)

Ethical considerations

The study protocol was reviewed and approved by the Scientific Board of St. Paul General Hospital of Thessaloniki (approval number: 6303469061-3ΑΘ). All procedures were performed in accordance with the ethical standards of the institutional research committee and the 1964 Declaration of Helsinki and its later amendments. Written informed consent was obtained from all participants prior to enrolment. No identifying information was collected, and all data were analyzed anonymously.

## Results

Participant characteristics

A total of 298 women participated in the study, with a mean age of 48.67 years (SD ± 10.66). Most participants resided in Thessaloniki (82.2%, n = 245), while 9.4% (n = 28) lived in rural areas and 8.4% (n = 25) in other cities. The majority were married (69.1%, n = 206), and educational attainment was high, with 43.3% (n = 129) holding a university degree and 18.1% (n = 54) reporting a postgraduate qualification. Detailed demographic characteristics are presented in Table 1.

**Table 1:** Demographic characteristics of study participants (N = 298) SD: standard deviation

Characteristic	n (%)	Mean ± SD
Residence		
Thessaloniki	245 (82.2)	-
Village	28 (9.4)	-
Other city	25 (8.4)	-
Educational level		
Primary	21 (7.0)	-
Secondary	85 (28.5)	-
University	129 (43.3)	-
Postgraduate	54 (18.1)	-
Doctorate	7 (2.3)	-
No response	2 (0.7)	-
Marital status		
Single	54 (18.1)	-
Married	206 (69.1)	-
Divorced	31 (10.4)	-
Widowed	6 (2.0)	-
No response	1 (0.3)	-
Age (years)	-	48.67 ± 10.66
Height (m)	-	1.65 ± 0.06
Weight (kg)	-	70.72 ± 15.89

Baseline psychosocial measures

At baseline, the mean General Self‑Efficacy score was 19.02 (SD ± 4.86), consistent with relatively low perceived self‑efficacy [8]. Baseline health anxiety was 10.99 (SD ± 5.35), indicating low to normal health-related anxiety. For the MHLC subscales, baseline means were as follows: internal control, 19.24 (±4.92); powerful others, 18.22 (±6.39); and chance, 14.35 (±5.40). Baseline psychosocial scores are summarized in Table 2.

**Table 2:** Baseline psychosocial measures SD: standard deviation, MHLC: Multidimensional Health Locus of Control

Subscale	Mean ± SD
General self-efficacy	19.02 ± 4.85
Health anxiety	10.99 ± 5.35
Internal control (MHLC)	19.24 ± 4.92
Powerful others (MHLC)	18.22 ± 6.39
Chance (MHLC)	14.35 ± 5.40

Temporal changes in psychosocial parameters

Self-Efficacy and Health Anxiety

Repeated measures analysis showed no statistically significant change in self‑efficacy across 12 months (baseline: 19.02 ± 4.86; six months: 18.72 ± 5.21; 12 months: 18.87 ± 4.96; F = 0.553, p = 0.570, ηp² = 0.002). No significant change was observed in health anxiety (baseline: 10.99 ± 5.35; six months: 11.03 ± 4.63; 12 months: 11.04 ± 5.10; F = 0.023, p = 0.955, ηp² = 0.000). Descriptive values are shown in Table 3 and inferential results in Table 4.

**Table 3:** Evolution of psychosocial scale scores over time SD: standard deviation, CI: confidence interval, MHLC: Multidimensional Health Locus of Control

Scale/subscale	Baseline mean ± SD	6 months mean ± SD	12 months mean ± SD	95% CI (12 months)
Self-efficacy	19.02 ± 4.85	18.72 ± 5.21	18.87 ± 4.96	18.30-19.44
Health anxiety	10.99 ± 5.35	11.03 ± 4.63	11.04 ± 5.10	10.46-11.62
Internal control (MHLC)	19.24 ± 4.92	18.73 ± 4.80	18.09 ± 4.58	17.56-18.61
Powerful others (MHLC)	18.22 ± 6.39	18.16 ± 5.60	17.51 ± 5.38	16.89-18.12
Chance (MHLC)	14.35 ± 5.40	14.75 ± 4.97	14.04 ± 4.97	13.47-14.61

**Table 4:** Repeated measures ANOVA for psychosocial scales (Greenhouse-Geisser correction) SD: standard deviation, ANOVA: analysis of variance

Scale/subscale	N	Baseline mean ± SD	6 months mean ± SD	12 months mean ± SD	F	df1(GG)	df2(GG)	p	ηp²
Self-efficacy	295	19.02 ± 4.86	18.72 ± 5.21	18.87 ± 4.96	0.553	1.93	568.80	0.570	0.002
Health anxiety	295	10.99 ± 5.35	11.03 ± 4.63	11.04 ± 5.10	0.023	1.59	466.89	0.955	0.000
Internal control (MHLC)	295	19.24 ± 4.92	18.73 ± 4.81	18.09 ± 4.58	9.496	1.84	540.44	<0.001	0.031
Powerful others (MHLC)	295	18.22 ± 6.39	18.16 ± 5.61	17.51 ± 5.38	3.443	1.69	496.66	0.040	0.012
Chance	295	14.35 ± 5.40	14.75 ± 4.97	14.04 ± 4.98	3.426	1.77	519.68	0.039	0.012

Health Locus of Control

Small but statistically significant declines were observed over time in internal control (baseline: 19.24 ± 4.92; 12 months: 18.09 ± 4.58; F = 9.496, p < 0.001, ηp² = 0.031). Similarly, powerful others (baseline: 18.22 ± 6.39; 12 months: 17.51 ± 5.38; F = 4.43, p = 0.040, ηp² = 0.012) and chance (baseline: 14.35 ± 5.40; 12 months: 14.04 ± 4.98; F = 3.426, p = 0.039, ηp² = 0.012) showed small but significant changes. Means are shown in Table 3, and RM‑ANOVA results are in Table 4.

Post‑hoc comparisons (Bonferroni‑adjusted) indicated that the reduction in internal control was significant from baseline to 12 months (p_adj < 0.001, Cohen’s dz ≈ 0.27), with smaller significant pairwise effects for the other MHLC subscales. Post hoc results are provided in Table 5.

**Table 5:** MHLC post hoc pairwise comparisons (Bonferroni adjusted) MHLC: Multidimensional Health Locus of Control

Internal control
Baseline vs 6 months: p_adj = 0.122, dz = 0.120
Baseline vs 12 months: p_adj < 0.001, dz = 0.265
6 months vs 12 months: p_adj = 0.085, dz = 0.128
Powerful others
Baseline vs 6 months: p_adj = 1.000, dz = 0.012
Baseline vs 12 months: p_adj = 0.042, dz = 0.144
6 months vs 12 months: p_adj = 0.121, dz = 0.120
Chance
Baseline vs 6 months: p_adj = 0.354, dz = −0.091
Baseline vs 12 months: p_adj = 0.746, dz = 0.067
6 months vs 12 months: p_adj = 0.047, dz = 0.142

Preventive Behaviors

The proportion of women reporting breast self‑examination increased markedly from 44.3% (n = 132) at baseline to 92.2% (n = 271) at six months and 85.0% (n = 250) at 12 months (Cochran’s Q = 188.96, p < 0.001) (Table 6).

**Table 6:** Proportion of women performing BSE over time BSE: breast self-examination

Time point	N	n (yes)	% (yes)
Baseline	298	132	44.3
6 months	294	271	92.2
12 months	294	250	85.0

Pairwise McNemar tests confirmed significant increases from baseline at both follow-up time points (p_adj < 0.001 for baseline vs. six months and baseline vs. 12 months), with a modest decline between 6 and 12 months (p_adj = 0.030). Pairwise results are provided in Table 7.

**Table 7:** Pairwise McNemar tests for BSE (Bonferroni adjusted) BSE: breast self-examination

Preventive behaviors over time points
Baseline vs 6 months: p_adj < 0.001
6 months vs 12 months: p_adj = 0.030
Baseline vs 12 months: p_adj < 0.001

The frequency of BSE practice also shifted toward more regular performance (Friedman χ² = 193.81, p < 0.001, Kendall’s W = 0.391), with significant increases from baseline to both follow-up assessments. Frequency analyses are provided in Table 8.

**Table 8:** BSE frequency (Friedman + post hoc Wilcoxon) BSE: breast self-examination

Frequency of BSE practice
Friedman χ² = 193.81, p < 0.001, W = 0.391
Baseline vs 6 months: p_adj < 0.001
Baseline vs 12 months: p_adj < 0.001
6 months vs 12 months: p_adj = 0.586

Rates of breast ultrasound increased slightly over time (baseline: 92.3%; six months: 96.9%; 12 months: 94.6%; Cochran’s Q = 14.00, p < 0.001), while mammography rates remained stable (baseline: 86.2%; six months: 89.4%; 12 months: 88.8%; Cochran’s Q = 5.25, p = 0.072). Screening behaviors are summarized in Table 9.

**Table 9:** Ultrasound and mammography rates over time

Complementary actions to BSE
Ultrasound: 92.3% → 96.9% → 94.6% (Q = 14.00, p < 0.001)
Mammography: 86.2% → 89.4% → 88.8% (Q = 5.25, p = 0.072)

Correlations between psychosocial variables

At six months, self‑efficacy showed a strong positive correlation with internal control (r = 0.43, p < 0.01). Health anxiety correlated positively with powerful others (r = 0.28, p < 0.01) and chance (r = 0.32, p < 0.01) (Table 10).

**Table 10:** Correlations between psychosocial variables at six months

Variable pair	Pearson’s r	p-value
Self-efficacy and internal	0.43	<0.01
Health anxiety and powerful others	0.28	<0.01
Health anxiety and chance	0.32	<0.01

Predictors of 12-month outcomes

Multiple regression analyses identified prior (six‑month) scores as the strongest predictors of 12‑month outcomes for all psychosocial variables (standardized β values ranging from 0.567 to 0.673; all p < 0.001). Age was a significant predictor of powerful others (β = 0.107, p = 0.040), and health anxiety at six months predicted higher chance attribution at 12 months (β = 0.102, p = 0.041) (Table 11).

**Table 11:** Predictors of 12-month psychosocial outcomes (multiple regression) MHLC: Multidimensional Health Locus of Control

Dependent variable (12 months)	Predictor (6 months)	β (standardized)	p-value
Self-efficacy	Self-efficacy	0.638	<0.001
Health anxiety	Health anxiety	0.636	<0.001
Internal control (MHLC)	Internal control (MHLC)	0.609	<0.001
Powerful others (MHLC)	Powerful others (MHLC)	0.673	<0.001
	Age	0.107	0.040
Chance (MHLC)	Chance (MHLC)	0.567	<0.001
	Health anxiety	0.102	0.041

Scale reliability

Internal consistency was high for all scales across time points. Cronbach’s alpha values for self‑efficacy and health anxiety exceeded 0.84 at all assessments. At the same time, the MHLC total showed good reliability (α = 0.816 at baseline), with subscale α ranging from 0.629 to 0.720 across time points (Table 12).

**Table 12:** Internal consistency (Cronbach’s α) of psychosocial scales MHLC: Multidimensional Health Locus of Control

Scale/subscale	Time point					
	Baseline		6 months		12 months	
Self-efficacy	0.869	297	0.848	285	0.884	287
Health anxiety	0.885	297	0.847	289	0.893	292
Total (MHLC)	0.816	293	0.774	279	0.816	274
Internal (MHLC)	0.665	294	0.641	286	0.643	288
Powerful others (MHLC)	0.688	293	0.629	289	0.663	285
Chance (MHLC)	0.720	294	0.634	283	0.682	289

## Discussion

The present study evaluated the impact of a structured BSE educational intervention on key psychosocial parameters among Greek women. The findings demonstrate that BSE education substantially increased the adoption and frequency of self-examination practices, without adversely affecting health anxiety or general self-efficacy. Small but statistically significant shifts were observed in health locus of control dimensions, suggesting nuanced adaptations in health beliefs over time.

The marked increase in BSE practice following education aligns with international evidence that structured interventions can effectively enhance preventive behaviors [6,7]. Systematic reviews have consistently shown that educational programs, particularly those incorporating practical demonstration, tailored materials, and motivational components, improve both knowledge and the likelihood of regular BSE [6,12]. The present study’s results reinforce the value of such interventions in populations with high baseline awareness but suboptimal engagement in preventive practices.

Despite the behavioral gains, the intervention did not significantly alter general self-efficacy or health anxiety. This stability is notable, as concerns have been raised that increased vigilance or ambiguous findings from self-examination could increase psychological distress [2,11]. The absence of higher anxiety in this cohort suggests that the educational approach, emphasizing empowerment, clear action steps, and appropriate follow-up, may mitigate the risk of excessive worry, a finding consistent with prior research [10,12]. This is particularly relevant given that international guidelines now prioritize breast self-awareness over routine BSE, aiming to balance early detection of abnormalities with minimization of harm [1,13].

The observed reductions in internal and external health locus of control subscales, though modest, may reflect a recalibration of health beliefs as participants integrate new knowledge and experience with the healthcare system. The decline in internal control suggests a shift from viewing health as solely a matter of personal agency toward recognizing the importance of professional evaluation and systemic support, a perspective increasingly emphasized in contemporary models of cancer screening and care [9,14]. The small decrease in attribution to “powerful others” and “chance” may indicate growing confidence in navigating the healthcare pathway, though the effect sizes were limited.

Regression analyses highlighted that prior psychosocial status was the strongest predictor of subsequent outcomes, underscoring the relative stability of these constructs over time. Age was associated with greater reliance on external authorities for health control, consistent with literature suggesting that older adults may defer more to healthcare professionals [9]. Additionally, higher health anxiety predicted increased attribution to chance, suggesting that persistent worry may be associated with more fatalistic attitudes, a dynamic warranting further exploration in future studies.

The findings must be interpreted in light of several limitations. The reliance on self-reported behaviors introduces potential for social desirability and recall bias. The study did not assess clinical endpoints such as stage at diagnosis or mortality, precluding conclusions about the direct impact of BSE education on breast cancer outcomes. Furthermore, the generalizability of the results may be limited to populations with similar access to healthcare and baseline levels of awareness. As emphasized by the World Health Organization, the effectiveness of breast health interventions depends on the existence of clear referral pathways and accessible diagnostic services [15].

Future research should consider objective assessment of BSE technique, stratification by risk group, and evaluation of care pathways from symptom recognition to diagnosis. Comparative studies of breast self-awareness versus structured BSE education, as well as the integration of digital or virtual learning modalities, may further clarify optimal strategies for promoting early detection without psychological harm [16,17].

In summary, this study supports the role of BSE education as a complementary measure for enhancing breast health awareness and preventive behavior. The intervention did not increase health anxiety or disrupt general self-efficacy, and minor shifts in health locus of control suggest adaptive changes in health beliefs. These results are consistent with current international recommendations, which position BSE education as an adjunct to, rather than a replacement for, organized screening programs [1,18,19]. Effective implementation requires clear communication of the limitations and appropriate context for BSE, ensuring that women are empowered to seek timely evaluation without undue psychological burden.

## Conclusions

This analysis demonstrates that structured BSE education significantly enhances preventive behavior among Greek women, with the proportion of women practicing regular BSE rising from nearly four out of 10 at baseline to over eight out of 10 at 12 months. Importantly, this behavioral change was not accompanied by increased health anxiety or alterations in general self-efficacy, indicating that the intervention was psychologically safe and well-tolerated. Minor but statistically significant reductions in internal health locus of control suggest a nuanced shift in health beliefs, reflecting greater integration of professional healthcare support alongside personal agency. These findings reinforce the value of BSE education as a complementary strategy for breast health awareness, supporting early care-seeking and engagement with healthcare services. However, the results do not advocate BSE as a substitute for formal screening; mammography remains the cornerstone of population-level breast cancer detection, as emphasized in current guidelines. The stability of psychosocial parameters and the absence of adverse effects highlight the potential for educational interventions to empower women without increasing psychological burden.

Limitations include reliance on self-reported behaviors, the potential for the Hawthorne effect, and the lack of clinical outcome data, which limits conclusions about direct effects on cancer detection or mortality. Future research should incorporate objective measures of BSE proficiency, stratify by risk group, and evaluate the impact on diagnostic timelines and clinical endpoints. Overall, BSE education can be recommended as an adjunct to organized screening to foster breast self-awareness and timely health-seeking, provided its role and limitations are clearly communicated within the broader context of evidence-based cancer prevention.
